# A universal tool for stability predictions of biotherapeutics, vaccines and in vitro diagnostic products

**DOI:** 10.1038/s41598-023-35870-6

**Published:** 2023-06-21

**Authors:** M. Huelsmeyer, D. Kuzman, M. Bončina, J. Martinez, C. Steinbrugger, J. Weusten, C. Calero-Rubio, W. Roche, B. Niederhaus, Y. VanHaelst, M. Hrynyk, P. Ballesta, H. Achard, S. Augusto, M. Guillois, C. Pszczolinski, M. Gerasimov, C. Neyra, D. Ponduri, S. Ramesh, D. Clénet

**Affiliations:** 1 Drug Product Development, AbbVie, Ludwigshafen, Germany; 2 Biologics Drug Product, Technical R&D, Global Drug Development, Novartis , Mengeš, Slovenia; 3 R&D Immunoassays, Biomolecule Engineering - bioMAP, bioMérieux, Marcy L’étoile, France; 4 Center for Mathematical Sciences, MSD, Oss, The Netherlands; 5 Biologics Drug Product Development & Manufacturing, Sanofi, Framingham, USA; 6 MSAT, Sanofi, Waterford, Ireland; 7 CMC-Biologics Statistics, Sanofi, Frankfurt, Germany; 8 CMC-Biologics Statistics, Sanofi, Gent, Germany; 9 Global Drug Product Bioprocess Development, Sanofi, Toronto, Canada; 10 Altran Technologies, Capgemini Engineering, Lyon, France; 11 Manufacturing Technology Department, Sanofi, Val-de-Reuil, France; 12 Manufacturing Technology Department, Sanofi, Swiftwater, USA; 13 Manufacturing Technology Department, Sanofi, Hyderabad, India; 14 Global Drug Product Bioprocess Development, Sanofi, Marcy L’étoile, France

**Keywords:** Biological techniques, Biotechnology, Computational biology and bioinformatics, Diseases, Health care, Oncology

## Abstract

It is of particular interest for biopharmaceutical companies developing and distributing fragile biomolecules to warrant the stability and activity of their products during long-term storage and shipment. In accordance with quality by design principles, advanced kinetic modeling (AKM) has been successfully used to predict long-term product shelf-life and relies on data from short-term accelerated stability studies that are used to generate Arrhenius-based kinetic models that can, in turn, be exploited for stability forecasts. The AKM methodology was evaluated through a cross-company perspective on stability modeling for key stability indicating attributes of different types of biotherapeutics, vaccines and biomolecules combined in in vitro diagnostic kits. It is demonstrated that stability predictions up to 3 years for products maintained under recommended storage conditions (2–8 °C) or for products that have experienced temperature excursions outside the cold-chain show excellent agreement with experimental real-time data, thus confirming AKM as a universal and reliable tool for stability predictions for a wide range of product types.

## Introduction

Shelf-life estimation methods, as described in the International Conference on Harmonisation (ICH) guidelines^1,2^ suggest using linear or nonlinear regression and statistical modeling through “poolability” tests. In practice, simple kinetics such as zero- or first-order mechanisms are typically used to estimate product degradation rates through accelerated stability programs which expose products to temperatures greater than those recommended (typically 5 °C, 25 °C, 37/40 °C). Even if though such approaches, designed for small molecules in mind, were already used with some success for complex bioproducts such as vaccines^3–5^, they very often fail to adequately describe the complex stability behaviour of bioproducts, which frequently involve complex and multi-step reactions^6,7^. This key issue can be overcome using advanced kinetic modeling (AKM) that considers linear, accelerated, decelerated and S-shaped kinetic profiles and their combinations. AKM provides phenomenological kinetic models able to well describe degradation rates of products, independently of their complexity of degradation pathways. While in certain cases the degradation rates of biotherapeutics and vaccines can be well-described with one-step kinetics^8–10^, two-step profiles are often required to describe complex degradations with an initial rapid drop followed by a long, gradual decrease stage as exemplified by numerous examples reported in literature^11–14^.

In the aim to develop the correct models, the use of “good modeling practices” in four stages were recommended for accelerated stability predictions of bioproducts^9,15,16^. The 1st stage defines rules to conduct an appropriate predictive stability study. It requires at least 20–30 experimental data points obtained with minimally three incubation temperatures (usually 5 °C, 25 °C and 37 °C or 40 °C). Additionally, a significant degradation (e.g., 20% of the ordinate Y-axis) should be reached under high temperature conditions and it should be larger than the one expected at the end of shelf life under recommended storage temperature. A screening of many kinetic models according to a least-squares regression analysis is required in the 2nd stage of good modeling practices. Simple models such as zero and first-order and more complex multi-step kinetic models must be screened to fit experimental accelerated stability data by systematic adjustment of kinetic parameters (i.e., *A*, *E*, *n*, *m*, see Eq. 1). The 3rd stage aims to the selection of the optimal model. The model best describing the reaction progress for the quality attribute under observation (i.e., loss of antigenicity, purity, etc.) is identified according to statistical parameters such as model comparison scores (AIC, Akaike information criteria; BIC, Bayesian information criteria), quality of fit (RSS, residual sum of square) and robustness of the fit (in the same model parameters should be obtained at different temperature intervals like 5–40 °C or 5–25 °C). The resulting reaction rate equation (kinetic model) can be used to simulate the reaction progress (e.g., degradation extent, emergence of decomposition products) over time for any chosen temperature profile (isothermal or fluctuating). The determination of the predictive bands (i.e., the prediction intervals at 95% or 99% level) of predictions is performed in the 4th stage by statistical analysis (e.g., bootstrap) of the experimental dataset.

This approach has been successfully applied for various types of vaccines (recombinant protein sub-unit, viral, glycoconjugates) and therapeutic mAbs coming from different companies^8,15^ and more recently on a polypeptide^17^.

In this work, AKM was utilized on real-life bioproducts including biotherapeutics, vaccines and in vitro diagnostic reagents. Comparisons of stability predictions with experimental data obtained for the products kept for long-term under recommended storage conditions or experiencing successive temperature excursions were done to verify accuracy of kinetic models. Based on literature data and the results presented in this work, AKM appears as a reliable approach for stability modeling and shelf-life prediction and should be encouraged as a routine practice to support the accelerated development of bioproducts. In this way, AKM can effectively reduce development risk by making stability data available (stability forecasts) at a very early stage of development what otherwise, would be available only at the very end of a procedure of stability evaluation.

## Materials and methods

Advanced kinetic modeling (AKM) that considers linear, accelerated, decelerated and S-shaped profiles was used as an appropriate solution to describe the reaction progresses of the selected stability attributes of a wide range of products developed in several companies (Table 1). Experimental stability data used in this study were generated with validated analytical methods.

**Table 1:** List of tested products, including biotherapeutics (B), vaccines (V) and in vitro diagnostics (D).

Type of product	Name/company	Format, presentation	Stability attributes	Comments
Biotherapeutics	B1/Abbvie	Liquid, mAb, IgG @ 150 mg/ml	Acidic isoforms	Supporting information for stability evaluation
Biotherapeutics	B2/Novartis	Liquid, mAb, IgG @ 10 mg/ml	Acidic variants, aggregates	Supporting information for stability evaluation
Biotherapeutics	B3/Sanofi	Liquid, mAb @ 1 mg/ml	Aspartate isomerization	Stability evaluation and commercial benchmarking
Biotherapeutics	B4/Sanofi	Liquid, mAb @ 150 mg/ml	Charged isoforms	Phase III product, modeling supporting dossier
Biotherapeutics	B5/Sanofi	Freeze-dried product, enzyme @ 4.2 mg/ml	Glass transition temperature (Tg), residual moisture content (RMC)	Supporting information for stability evaluation
Biotherapeutics	B6/Abbvie	Liquid, mAb @ 40 g/L	Monomer content (SEC)	Temperature excursion at end of shelf life
Biotherapeutics	B7/Sanofi	Liquid, single variable domain @ up to 150 g/L	HMW % (SEC)	Concentration dependent shelf-life estimation
Biotherapeutics	B8/MSD	Liquid, mAb @ 25 g/L	The emergence of impurity %	Shelf-life estimation
Biotherapeutics	B9/Novartis	Liquid, fusion protein, @ 40 mg/ml	Purity (rCE-SDS)	Supporting information for stability evaluation
Biotherapeutics	B10/Novartis	Liquid, fusion protein, @ 50 g/L	Aggregates HMW(SEC)	Supporting information for stability evaluation
Biotherapeutics	B11/Novartis	Liquid, mAb @ 50 mg/mL	Aggregates HMW (SEC)	Supporting information for stability evaluation
Bulk vaccine	V1/Sanofi	Frozen liquid, bacteria-based working seed lot, commercial product	Cell viability	Supporting information for shelf-life evaluation
Multivalent vaccine	V2/Sanofi	Full liquid	Depolymerization (%)	Shelf-life estimation
Inactivated vaccine	V3/Sanofi	Liquid, splitted virus	Antigen content	Shelf-life estimation
Polysaccharide-Protein conjugate vaccine (MenQuadfi)	V4/Sanofi	Multivalent polysaccharide, liquid	Free polysaccharide (%)	Impact of successive excursions of temperature. Batch-to-batch comparison
Live-attenuated virus	V5/Sanofi	Freeze-dried product	Infectious titer (CCID50)	Impact of successive excursions of temperature
Quadrivalent vaccine	V6/Sanofi	Liquid, adjuvanted, commercial product	Antigenicity of antigen A and antigen B (ELISA)	Impact of successive excursions of temperature
Live-attenuated virus	V7/Sanofi	Freeze-dried commercial product	Infectious titer (CCID50)	Impact of successive excursions of temperature
Live-attenuated virus	V8/Sanofi	Freeze-dried product	Infectious titer (CCID50)	Impact of successive excursions of temperature
In vitro diagnostic—VIDAS PTH (1–84)	D1/bioMérieux	Full kit (i.e., standard vial S1, control vial C1, coated SPR, strips, except 4-MUP fluorescent substrate)	VIDAS relative fluorescent value on Control C1 vial	Shelf-life estimation
In vitro diagnostic—VIDAS Cortisol S	D2/bioMérieux	Full kit (i.e., standard vial S1, control vial C1, coated SPR, strips, except 4-MUP fluorescent substrate)	VIDAS relative fluorescent value on Standard S1 vial	Shelf-life estimation
In vitro diagnostic—VIDAS NEPHROCHECK	D3/bioMérieux	Control vial C1 (IGFBP7), liquid	VIDAS relative fluorescent value on Control C1 vial	Shelf-life estimation
In vitro diagnostic—VIDAS NEPHROCHECK	D4/bioMérieux	Control vial C1 (TIMP-2), liquid	VIDAS relative fluorescent value on Control C1 vial	Shelf-life estimation

AKM can consider from the simplest towards the most complex degradation pathways of products^13^, supplying phenomenological kinetic models defined by reaction rates $\frac{d\alpha }{dt}$ of evaluation of stability attributes. The most complex degradations can be described as the sum of individual one-step reactions in the form of a competitive two-step kinetic:

1 $$\begin{gathered} \frac{d\alpha }{{dt}} = v \times A_{1} \times \exp \left( { - \frac{Ea1}{{RT}}} \right) \times \left( {1 - \alpha_{1} } \right)^{n1} \times \alpha_{1}^{m1} \times C^{p1} \hfill \\ \quad \quad \;\;\; + \left( {1 - v} \right) \times A_{2} \times \exp \left( { - \frac{Ea2}{{RT}}} \right) \times \left( {1 - \alpha_{2} } \right)^{n2} \times \alpha_{2}^{m2} \times C^{p2} \hfill \\ \end{gathered}$$

with the pre-exponential factor $A$, the activation energy ${E}_{\alpha }$, the order of the reaction *n* and a parameter *m* accounting for a possible autocatalytic-type contribution of the reaction. ${\varvec{v}}$ *ratio*, describing the contribution of the first reaction to the total rate of degradation path, R is the universal gas constant, T the temperature in Kelvin and *C*, the concentration of proteins at the start of the reaction and *p*, the associated fitted number. ${C}^{p}$ was specifically introduced and used only for single variable-domain (B7, Table 1), the terms *C* and *p* being related to the influence of the concentration of protein on the degradation rate.

In practice, the development of such Arrhenius-based models is done by optimization iterations of the kinetic parameters to fit the real experimental stability data obtained at different temperatures, including recommended storage (5 °C ± 3 °C) and accelerated conditions (e.g., 25 °C and 37 °C or 40 °C). Applying good modeling practices^9,15,16^, various kinetic models, from simple to more sophisticated ones, are screened to fit short-term accelerated stability data. The simplest model best describing the progress of the change of considered attribute is then selected using statistical scores such as (corrected) Akaike and Bayesian Information Criteria (AIC/BIC)^18^. In a few cases where AIC/BIC criteria disagreed, several ‘best models’ were considered to perform multiple bootstraps on each model ranking them by AIC and BIC weighted scores to obtain realistic predictions. Using this multiple model bootstrap (MMB) approach, bootstrap was made on a single model, with a number of loops $({N}_{loops})$ being proportional to its respective values of the weights wAIC and wBIC. The AKTS-Thermokinetics software^19^ (version 5.5) was used to perform AKM and associated stability predictions. Alternatively, SAS^20^ (version 9.4) was used for stability modeling of mAb B8. For comparison, classical linear regressions of stability data obtained under recommended storage conditions^2^ were performed using JMP^21^ (version 16).

Shelf-life Cards (AKTS) were used to monitor during the transport or storage of vaccines the variations of temperature, humidity, luminosity, and geolocation in real-time of vaccines experiencing temperature excursions. As described above, the previously created kinetic models, are combined with the Shelf-life Cards (SLC) through the AKTS data management software solution. The advanced kinetic analysis combined with the SLCs allows continuous monitoring of the level of vaccine degradation within the management software.

All kinetic models are listed in Supplementary information section.

## Results

Various examples of accelerated stability studies were collected, covering a wide range of applications, including screening of formulations, estimation of shelf-life and impact of an excursion of temperature at the end of shelf-life (in-use conditions).

### Stability Predictions of Biotherapeutics 

**Figure 1:**
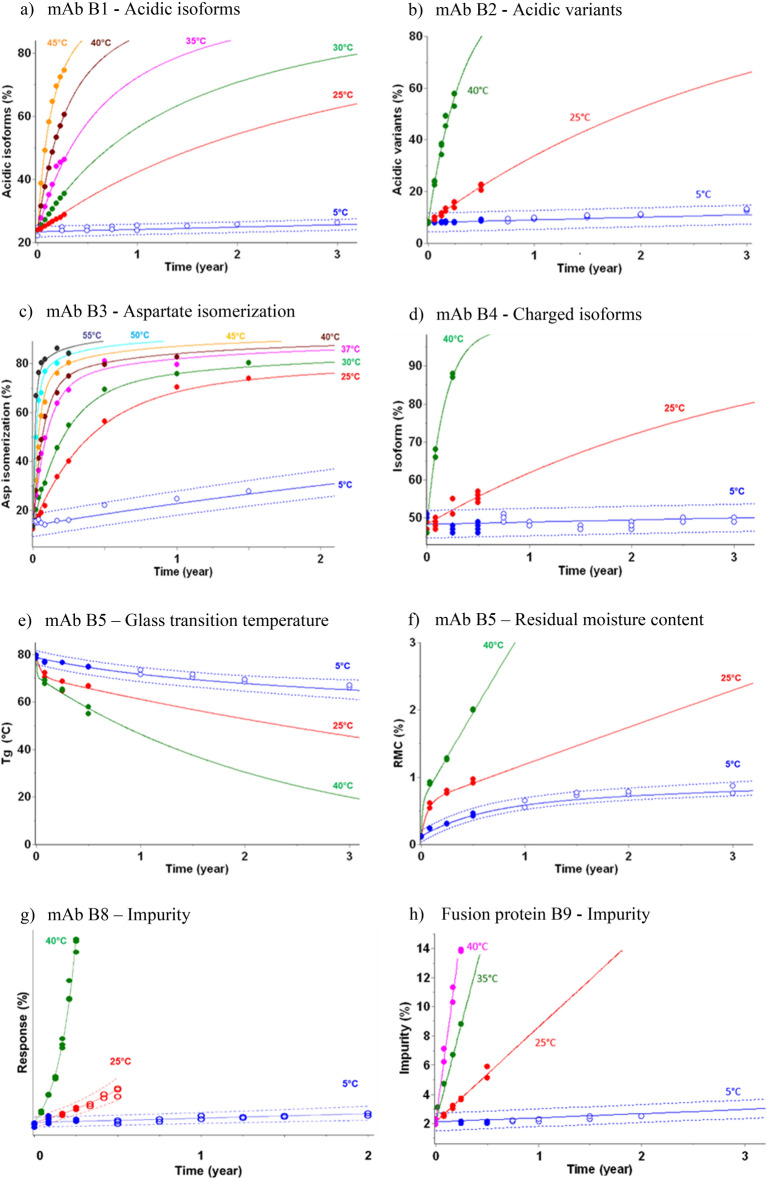
Kinetic modeling of biotherapeutics in liquid and freeze-dried pharmaceutical forms—Stability predictions for key quality attributes of various biotherapeutics—(**a**) Acidic isoforms (mAb B1), (**b**) Acidic variants (mAb B2), (**c**) Aspartate isomerization (mAb B3), (**d**) Charged isoforms (mAb B4), (**e**) Glass transition temperature (mAb B5), (**f**) Residual moisture content (mAb B5). Data points (filed circles) from 5 °C to 55 °C used to create the kinetic models and associated predictions (solid lines) are displayed. The prediction at 5 °C is shown as predictive bands representing the 95% PB (dotted lines). Additional experimental stability data (open circles), not used to build the models, are displayed to verify the stability predictions.

Various stability attributes were monitored during accelerated stability studies up to 40 °C, 45 °C or 55 °C for three or six months for various mAbs (Fig. 1a–g) and one fusion protein (Fig. 1h). In all cases kinetic models led to stability predictions in full agreement with real-time stability data at 5 °C up to 3 years.

**Figure 2:**
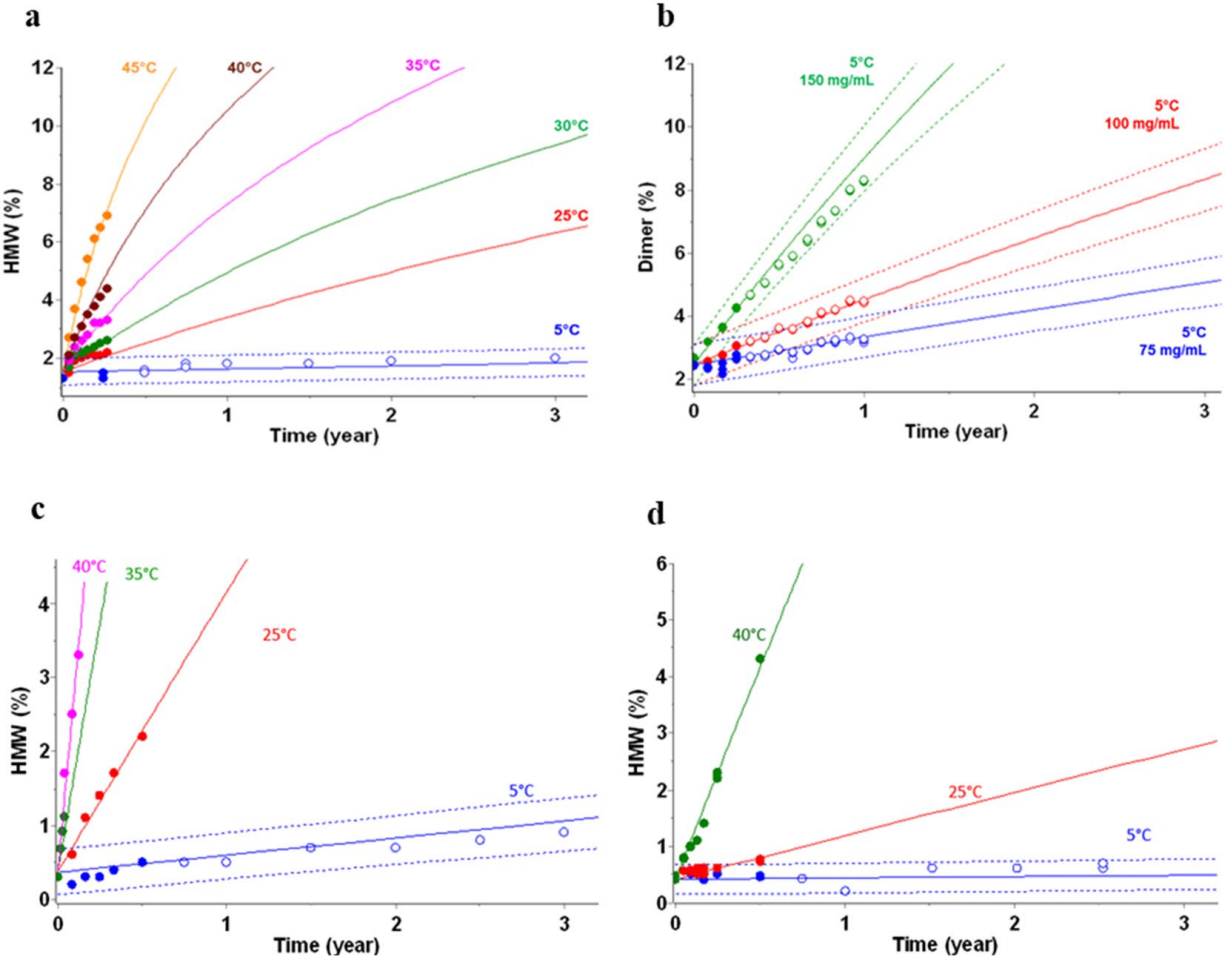
Kinetic modeling of aggregation of biotherapeutics—Stability predictions for several bioproducts—(**a**) HMW (mAb B1), (**b**) dimer (Single variable domain B7), (**c**) HMW (Fusion protein B10), (**d**) HMW (mAb B11). Data points (filled circles) from 5 °C to 45 °C used to build kinetic models overlaid with predictions (solid lines). The prediction is shown as predictive band representing the 95% PB (dotted lines). Additional stability data (open circles), not used to build the models, are displayed as verification of the stability predictions.

Aggregation, considered as a main degradation pathway of protein in solution, was monitored during accelerated stability studies up to 45 °C for three or six months for various biotherapeutics, including mAbs (Fig. 2a and d), a single variable domain (Fig. 2b) and a fusion protein (Fig. 2c). For the latter, the use of data collected up to 50 °C led to inaccurate prediction at 5 °C (not shown). Restricting the modelling to data in the 5–40 °C temperature range led to an accurate prediction. This is aligned with good modeling practices recommending development of kinetic models using data collected in a temperature range assuring that the degradation path is not changing^10,16^. Following the rule, HMW and dimer formation predictions agree with long-term experimental data at 5 °C.

### Stability predictions of vaccines

**Figure 3:**
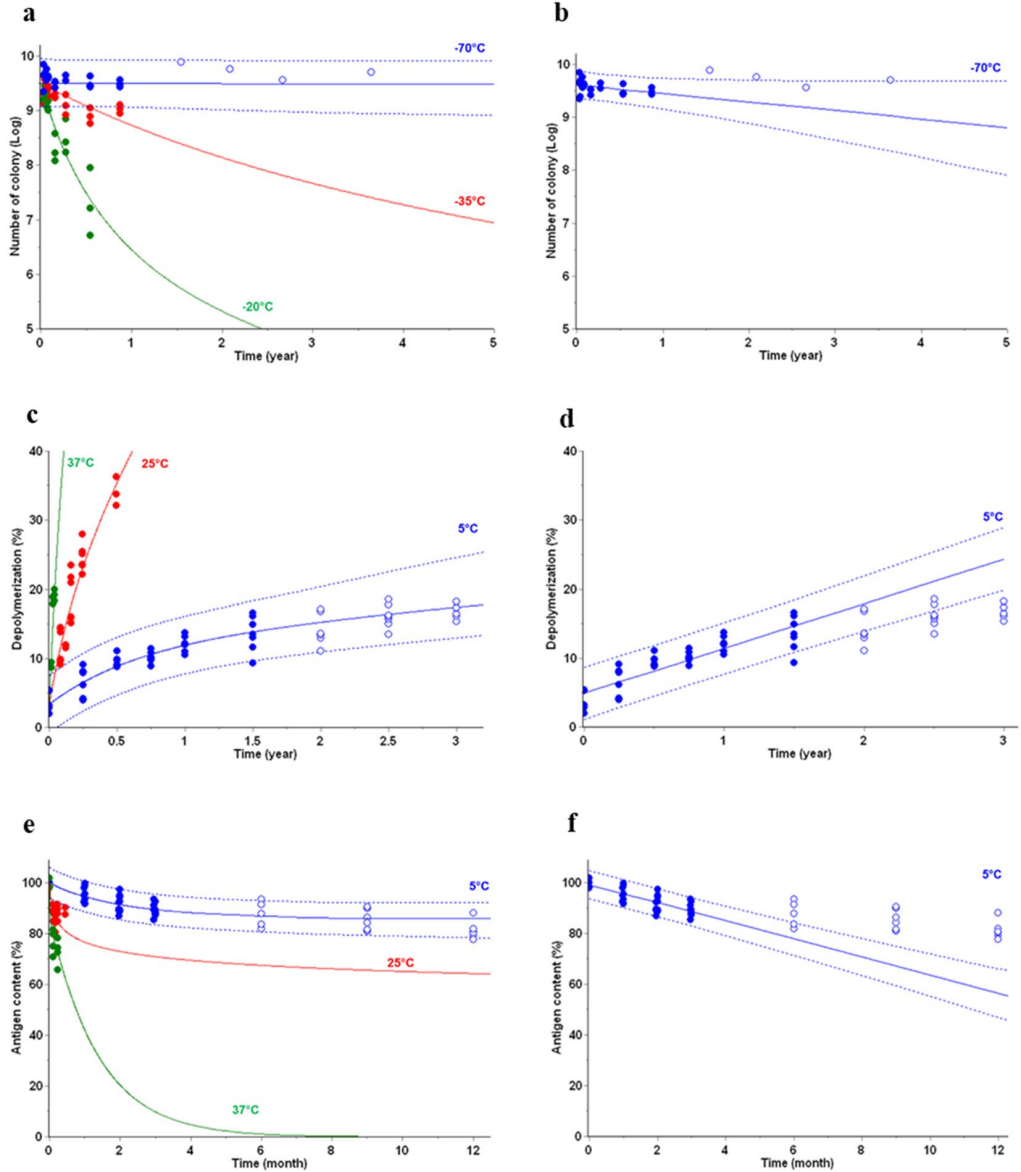
Vaccines stored under recommended storage conditions—Comparison of AKM (left column) using recommended and accelerated storage temperatures *vs*. linear regression ICH-based method (right column) using the recommended storage temperature, only (i.e. − 70 °C or + 5 °C)—(**a**–**b**) Cell viability (Bulk vaccine V1), (**c**–**d**) Depolymerization (Vaccine V2), (**e**–**f**) Antigen content (Vaccine V3). Data points (filled circles) used to build kinetic models overlaid with predictions (solid lines). The prediction is shown as predictive band representing the 95% PB (dotted lines). Additional stability data (open circles), not used to build the models, are displayed as verification of the stability predictions.

Different types of vaccines, including one bulk under frozen state were chosen to apply AKM, *vs*. ICH-based method, then evaluate accuracy of predictions of three different key stability indicating attributes. 3 temperatures were used to develop kinetic models by AKM (Fig. 3—left column), while only 5 °C stability data were used for linear regression as proposed by ICH guideline^1^ (Fig. 3—right column). In all cases, kinetic models developed by AKM led to the stability predictions under recommended storage conditions (i.e. − 70 °C or + 5 °C) showing full agreement with experimental long-term stability data. In contrast, ICH-based method led to wrong predictions compared with the same experimental long-term stability data, missing evident curvature required to accurately describe reaction progresses of the three selected attributes.

### Stability predictions of immunoassay in vitro diagnostic

**Figure 4:**
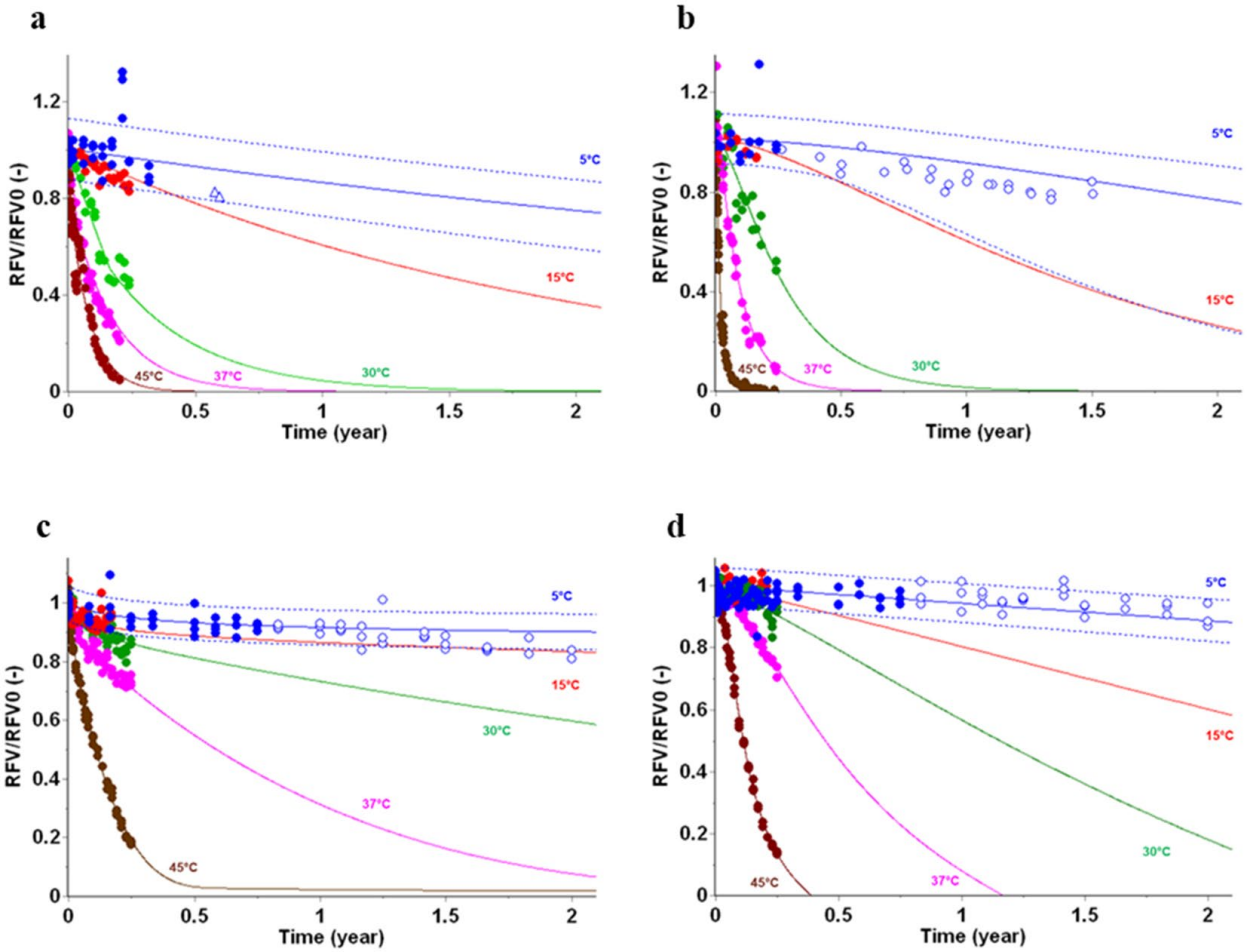
Stability predictions for key attributes of an in vitro diagnostic full kits—(**a**) VIDAS Cortisol S, CORS, D2, (**b**) VIDAS PTH C1 STAB, 1-84, D1 using different batches of kits, (**c**) VIDAS NEPHROCHECK - C1 IGFBP-7 D3, (**d**) VIDAS NEPHROCHECK - C1 TIMP-2 NC, PTB7 D4 using a single batch of Calibrator (C1). Data points (filled circles) from 5 °C to 45 °C expressed as RFV (Relative Fluorescence Value) and used to build kinetic models for key stability attributes of these in vitro diagnostic kits or reagents are overlaid with predictions (solid lines). The prediction at 5 °C is shown as predictive bands representing 95% limit (dotted lines). Additional stability data (open symbols), not used to build the models, are displayed as verification of the stability predictions.

An automated solution of ELFA based immunoassays (VIDAS, cf. Suppl. Mat. for details) was used to evaluate performance of AKM. Figure 4a shows an AKM performed with VIDAS Cortisol S reagent, 14 years after the real time stability study. Despite of different batches and time gap between both studies, and of historical poor real time data collection (only at 7.2 months), this study demonstrates that real time stability results at 5 °C are covered in the predictive band of the kinetic model.

Figure 4b shows an AKM prediction realized using VIDAS PTH kit with a 3-months data collected from 5 °C to 45 °C overlaid with 18 months of real-time stability of 2 other different batches. In that case, the real-time data of both batches fits with the predicted model despite a broadening of the PB bands during time.

Figure 4c, d show a study performed during the late stage of development of VIDAS NEPHROCHECK with the opportunity of testing a same batch for both AKM (9 months of discontinuous data collection) and real-time stability follow-up. The mean values of real time stability results at 5 °C are mainly included in the predictive band of the kinetic models up to 2 years.

### Batch comparison to support product development and post-approval changes

**Figure 5:**
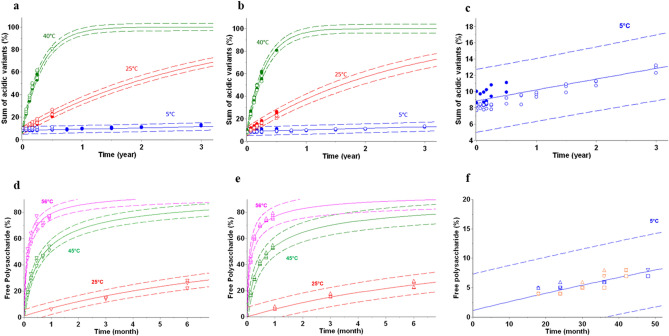
Batch-to-batch comparisons—Stability predictions for key attributes of different batches of a biotherapetic (mAb B2—panels **a**–**c**) and a vaccine (vaccine V4—panels **d**–**f**). The predictions (solid lines) are shown as predictive band representing the 99% PB (dashed lines), overlayed with stability data (symbols). Form mAb B2, experimental stability data (filled circles) of three technical batches (panel **a**) and three clinical batches (panel **b**) were used to develop kinetic models and associated predictions (lines). Open circles represent experimental data of clinical (panel **a**) and technical batches (panel **b**) and are displayed as verification of the stability predictions. For vaccine V4, kinetic models and associated predictions (lines) of small scale (panel **e**) and large scale (panel **d**) batches are overlaid with experimental stability data (open symbols) of large scale (panel **e**) and small scale (panel **d**) batches. Cross comparisons of experimental data and model predictions are shown in panel (**c**) and (**f**), for mAb B2 and vaccine V4, respectively.

AKM was used to compare stability of batches. For mAb B2, using the sum of acidic variants as stability critical quality attribute, pilot technical batch and clinical batch are compared, formulation and primary packaging being identical. Early development multi years stability data (Fig. 5a) can be used to verify models build on late-stage data where usually long-term stability data is not available (Fig. 5b). Verification of the clinical batch model was achieved by comparing associated predictions at 5 °C with real stability data of the technical batch (Fig. 5c).

In glycoconjugate vaccines, the term free (poly)saccharide refers to the amount or proportion of (poly)saccharide that is present but not covalently attached to the protein carrier. This is an important attribute because only polysaccharide directly linked to the carrier protein, i.e., conjugated polysaccharide, can afford and sustain complete clinical protection. Free polysaccharide is generated through the hydrolysis of labile acetal linkages and is a sensitive stability-indicating parameter. AKM was applied to the Serogroup ACWY Quadrivalent MenQuadfi Polysaccharide-Protein conjugate vaccine stability data to identify kinetic models which describe an increase in free polysaccharide for two production scales (tens of liters and hundreds of liters) of this vaccine. Based on 6-months stability data at 25 °C, 45 °C and 56 °C kinetic models suggest that both production scales generated products with comparable thermal stability (Fig. 5d and e). Furthermore, using the kinetic model developed for small scale, long-term stability (i.e., up to 4 years) at 5 °C were accurately predicted for both scales batches (Fig. 5f).

### Stability predictions for products experiencing temperature excursions

**Figure 6:**
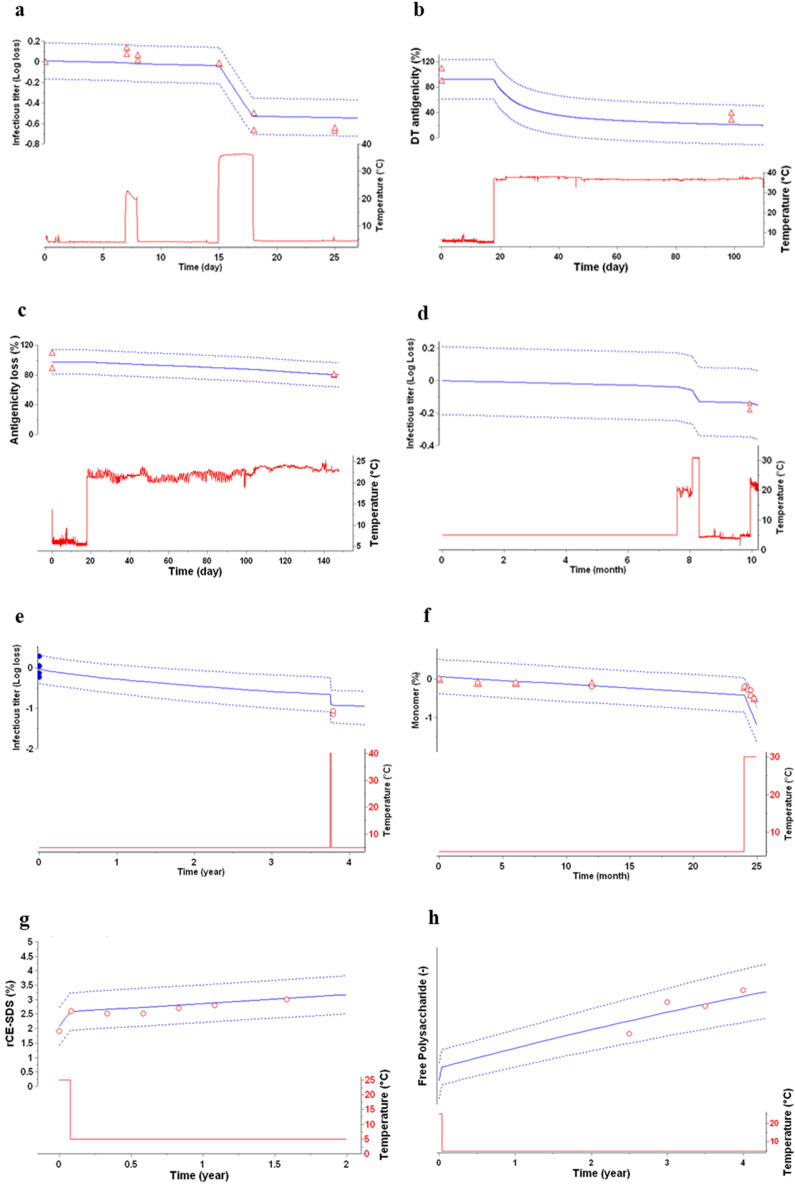
Prediction model for cold chain breaks of drug products—Stability predictions (blue lines) in real time for various bioproducts during storage including temperature excursions outside recommended conditions (bottom part—red lines). The real-time predictions are shown with predictive band representing the 95% PB (top part—dotted blue lines). Additional stability data (open red symbols), not used to build the models, are displayed as verification of the stability predictions. (**a**) Infectious titer (Vaccine V5), (**b**) Antigenicity of antigen A (Vaccine V6), (**c**) Antigenicity of antigen B (Vaccine V6), (**d**) Infectious titer (Vaccine V7), (**e**) Infectious titer (Vaccine V8), (**f**) Monomer (mAb B6), (**g**) Impurity (Fusion protein B9), (**h**) Free polysaccharide (Vaccine V4).

Constituting fingerprints of thermal stability of products, kinetic models can be used to monitor in real-time the quality of products during their storage and shipments, continuously transforming temperature fluctuations recorded by an electronic device in degradation progress of products^22^. For six different vaccines, one mAb (B6) and one fusion protein (B9), kinetic models of key stability attributes were developed and then used to monitor the level of degradation considering their respective storage conditions (Fig. 6a–f). For live-attenuated vaccines (V5, V7 and V8, Fig. 6a, d and e, respectively), experimental data looked in agreement with progresses of the infectious titres monitored over the storage period including few days excursions from ambient temperature up to 40 °C. Same accurate predictions were obtained with other types of vaccines (V6 and V4), considering antigenicity or free polysaccharide as key attributes (Fig. 6b, c, h). Finally, full agreement between predictions and experimental data (purity level) were also observed for both biotherapeutics (B6 and B9) that suffered a short cold chain break at t-zero or at the end of shelf-life (Fig. 6f, g).

## Discussion

In recent years, simple models such as first-order kinetics with single-step reaction provided a consistent framework to describe the changes of critical attributes for bioproducts^5,8,17^. However, in some cases more complex two-step models were required to accurately predict stability progression of key attributes of bioproducts^12,13,17,23,24^. Both single and two-step models are comprised in so called advanced kinetics modeling (AKM). AKM enables to go beyond the ICH and WHO recommendations for stability predictions of products^25^ and has found successful application in development of biotherapeutics and vaccines^8,15,17,24^.

Kinetic based models developed using stability data at different temperatures are found to provide a significantly more accurate approximation when compared to the linear regression models of stability data at only one temperature (Fig. 3) typically used in classical stability modeling (based on ICH Q1E). For vaccine V2, the linear regression model underfits the data from the later stage of the study where the depolymerization rates starts to level off. Resultantly, shelf-life of the product, expected at 3 years at 5 °C, would be needlessly restricted if a linear regression model is used for stability evaluation. Same conclusion was reached considering vaccine V3, with an expected shelf-life at 12 months at 5 °C, not conveniently predicted since linear regression is applied. Another limitation of the classical approach is that they are based only on long-term stability data, which takes long time to generate. Whereas, by using data generated at different temperatures, the kinetics-based approach improves prediction accuracy and reduces the amount of data and time required.

This work is a compilation of multiple stability programs from early development to industrialization, and from different commercial entities that follow exclusive pharmaceutical development strategies. The number of points (time) used to develop kinetic models is aligned with good modelling practices with at least 20–30 experimental data points obtained with minimally three incubation temperatures (usually 5 °C, 25 °C and 37 °C or 40 °C). Furthermore, there is no reason to collect the points in strongly restricted time intervals. Modern advanced kinetic analysis allows flexible data collection. The main requirement for the correct kinetic analysis lies in the experimental points’ amount but not in their distribution in the coordinates: time-reaction extent.

The presented examples from a diverse bioproduct spectrum illustrate that AKM represents a very valuable tool in product development and stability evaluation. Its versatility offers multiple application possibilities in the pharmaceutical industry: AKM can be used to model and predict a wide range of quality attributes, irrespective of product type or molecule (biopharmaceutic, vaccine, diagnostic proteins). In addition, AKM is amenable to multiple presentation formats such as frozen state, liquid and lyophilisate. There are also no limitations in usable analytical methods: In principle, any measurable change of a quality attribute induced by a temperature shift can be utilized. As a new field of application, one can refer to gene therapy approaches such as AAVs and mRNA-based product candidates, recently tested in the clinic for several therapeutic and prophylactic indications. Due to their high thermosensitivity, this new class of products requires drastic storage conditions (i.e. storage under cold and ultra-cold conditions) to ensure their stability from production to use^26,27^. It can be assumed that AKM can support the determination of shelf-life and appropriate storage temperature helping to quantify the degree to which temperature excursions impact product quality and can also reduce product wastage. Furthermore, stability modeling would be of high interest to accelerate and support development of next generation more stable formulations.

The versatility of AKM does not come at the cost of accuracy. The examples in this publication establish that the mathematical procedures are mature and robust to deliver reliable results. However, the importance of proper temperature condition selection should not be underestimated, considering that development of kinetic models using data obtained at too high temperature can lead to erroneous predictions. The use of reasonable temperature range assuring that the degradation path is not changing is part of good modelling practices^16^ and was already highlighted specifically for aggregation of mAb^10^, advocating to limit the highest incubation temperature well below denaturation temperature (Tm) of the considered protein.

Prior knowledge or also termed risk-based modeling approaches (Risk Based Predictive Stability (RBPS)) are quite common in the pharmaceutical industry and a recent survey revealed that 55% of the companies using RBPS tools leverage these data in a regular context^28^. However, if prior knowledge is lacking, what is not unusual with the ever-increasing number of new molecule formats, AKM can tap to the full potential: Advanced kinetic modeling represents a *first principles* approach that is independent from any prior knowledge. An un-biased view on stability evaluations has clear benefits and due to its versatility, we encourage developers to also use the AKM approach for well-known product classes. The formation of aggregates exemplifies the notion: Intensively studied by the life-science community it has been theorized to be a multi-pathway process, gave rise to highly complex kinetic models and often appeared to have non-Arrhenius-like temperature dependencies described as a sum of reactions with different energetic barriers^29^. Missing (mechanistical) knowledge is not hampering the AKM approach: The phenomenological mathematical model that is derived for fitting the reaction progress, without any mechanistic basis, readily allows modeling processes that show Arrhenius and non-Arrhenius behavior and accurately predict stability progression. Furthermore, to consider the impact of the concentration of the single variable domain on the aggregation rate, two additional terms C and p can be added to such kinetic model (Eq. 1), as exemplified in Fig. 2b, describing the emergence of dimers as function of time and concentrations of a single variable domain, when stored at 5 °C.

Temperature excursion (TE) studies play an increasingly important role in product development. This trend illustrates the awareness of the pharmaceutical industry that products experience temperature changes under real-life conditions that could impact product quality. Thermal fluctuations during storage and transit can provoke partial denaturation, aggregation and chemical degradation of proteins and render them ineffective^30^. Failures in cold chain have a substantial negative impact for quality of bioproducts, considering that they must be kept refrigerated from production to use^31^. It is especially critical for vaccines, as the cold chain in the last mile is particularly demanding^32–34^. AKM can advantageously be used to predict in real-time and using real temperature fluctuations experiences by products, the impact of any kind of storage conditions, including temperature excursions, independent of their complexity provided that temperature was accurately recorded and used for prediction^22,24^.

In addition, some regulatory authorities (TGA Australia, Anvisa Brazil) request TE studies with the same rational in mind. To cover more than three years of incubation time (temperature cycling plus recommended storage conditions (RSC)) such studies are lengthy and elaborate. As illustrated in the presented examples AKM is capable to predict the progression of quality attributes for any type of temperature profile (isothermal, steps, complex) making stability statements accessible within weeks or months what under experimental real-time conditions would take years to obtain. In addition, an experimental TE study delivers data for exactly one temperature profile, while using AKM numerous scenarios can be simulated. For obvious reasons the latter situation is better suited to cover a plethora of real-life conditions for the benefit of the patient that is allowed, as an example, to store a medication at room temperature for a defined period instead of cooled without compromising its quality.

Manufacturing of bioproducts can also benefit from AKM assisted stability predictions as these processes often take place under non-RSC at elevated temperatures what could be described as temperature excursion. The faster formation of degradation products (e.g., high molecular weight species) can easily be calculated for the definition of an admitted maximal residence time at these conditions covering planned and unplanned events during manufacturing (hold-time definition). For stable biopharmaceuticals such as therapeutic antibodies it must be noted that the degradation speed is nevertheless very slow, so that accurate analytic analysis is prohibited. As an example, based on the kinetic model, HMW formation for mAb1 (Fig. 2a) at 25 °C can be calculated to be 0.0046% per day or 0.032% HMW per week. A direct analytical measurement is impossible as SEC is unable to detect these tiny changes. AKM makes these values accessible allowing a criticality assessment for product quality in accordance with QbD principles.

To make the impact of AKM tangible, two Sanofi vaccines may serve as examples. First, using kinetic models with the prediction data up to 48 months at 2–8 °C (recommended storage condition) for a rabies vaccine, it was possible to extend shelf-life for a clinical batch to secure Phase III evaluation. Not only could the models show that this product can withstand successive temperature excursions at 25 °C without impact on its main quality attribute and provide and justify the shelf-life claim, the use of AKM resulted in a significant savings in development time in addition to substantial financial benefits for the manufacturer. Second, as illustrated in Fig. 6h, AKM allowed to be confident in the quality of MenQuadfi commercialized vaccine with temperature excursions until expiry. These models were included to support a MenQuadfi time out of refrigeration claim for the end users and approved by several health authorities.

Batch comparison study is usually performed to support product development and post-approval changes: According to the current EMA guidance^35^ the maximum shelf-life of investigational medicinal product (IMP) after the extension should not be more than double, or more than twelve months longer than the period covered by real time stability data obtained with representative batch(es). Reliable and verified models to predict degradation profile of limiting quality attributes are tools that could be used to extrapolate shelf life even beyond currently accepted limits. One could claim 18 or even 24 months of clinical batch shelf life, based on kinetic model derived from 6 months accelerated stability (Fig. 5b). The only way to verify the prediction model is by using prior data from representative drug product stability study (Fig. 5a). As shown on Fig. 5c a modelled degradation rate and activation energy of clinical batch are akin to that obtained by technical batch model. Model parameters indicate that both degradation processes are comparable, and that real data verification of technical batch model can be attributed to the stability prediction of clinical batch. However, validity of the clinical batch model is further continuously monitored by future pull point data. In this context, within a comparably short time frame (weeks to few months) and manageable experimental effort reliable stability predictions for years are possible. Assuming stability data collected at different temperatures for at least three representative batches are available, these data can be pooled and used to generate a “generic kinetic model” for the considered product. Then predictive bands from this model can define fingerprints of thermal stability of the product and can be used for stability trending studies for further batches, comparing experimental stability data with predictive bands. Another way to compare batches can be proposed by treating AKM coefficients (i.e., kinetic parameters) amenable to batch variability as random batch effects (e.g., treating the lot-to-lot differences as coming from a population distribution, from which a range of degradation variability encompassing current and future batches can be inferred). Modeling of random lot effects are well described for linear single storage condition stability regressions^36^ and an implementation of this principle to the AKM approach was recently introduced^37^. Given the CMC importance of potential product variability during shipment and storage, the subject deserves further deepening.

Taken together, leveraging stability modeling during CMC development. During pre-clinical development stages of Drug Products, limited information is known around Critical Quality Attributes (CQAs) and the overall shelf-life of the clinical Drug Product. Moreover, development timelines are constantly challenged with the aim to (1) reduce overall development costs of new therapies and (2) accelerate timelines as CMC usually remains on the critical path to a Phase 1 regulatory filing^38–40^. As such, there is no opportunity to validate shelf-life of the Drug Product during development and accelerated studies at 25–40 °C are instead standard practice to identify CQAs with varied capacity to estimate recommended storage shelf-life^41–44^. Decision making during early stages is thus mostly based on a risk assessment and the likelihood of achieving a desired shelf-life instead of a real-time measurement. This is of higher relevance on the on-going transition from Intravenous-only therapies to subcutaneous and more patient-friendly routes of administration of biotherapeutics in increasingly competitive clinical trials, necessitating the development of liquid and high concentration Drug Products.

Results presented in this report show that properly designed accelerated studies coupled with kinetic modeling can accurately predict shelf-life of several degradation pathways without any a priori knowledge of the underlying mechanism. As such, and within the limited time granted to CMC development, prediction of shelf-life based on accelerated studies can become a potent tool to reduce the risk during decision making, especially during formulation selection (if shelf-life is expected to be impacted) and the determination of critical process parameters (CPPs). Additionally, these can be achieved with minor modification to development workflows (i.e., low resource investment) and within the recommendations of ICH Q8 guidelines. In this contact, updates to ICH Q1 and Q5C Stability Guidelines can be considered. Recently, focusing on vaccines, Campa provided an overview of cross-company discussions and dialogue with regulators around update of ICH guidelines^45^, the use of prior and platform knowledge, along with the use of predictive stability modeling are considered key enablers of stability assessment in accelerated scenarios. Results presented in our paper aligned with the best practices for modeling for vaccines, presented at the COVAX workshop^46^. It was highlighted that advanced-kinetic modeling makes it possible to go beyond the current ICH and WHO recommendations for stability predictions of products.

In practice, AKM has already been used to simulate the stability of several parameters for a wide range of vaccines and has already been presented to several National Regulatory Authorities (such as BGTD in Canada, ANSM in France, MEB in Sweden, TGA in Australia, COFEPRIS in Mexico, EMA in Europe) to support new stability claims for products with positive outcomes.

## Conclusions

Stability studies are an indispensable part of all development activities. For bioproducts this is often challenging given the complexity of their degradation paths and facing short development timelines. This work illustrates how advanced kinetic modeling can be used as a universal tool that can not only be applied for stability forecasts but is also useful across the entire product development and lifetime, during formulation development and analytical comparability studies, in characterization of temperature excursions, post-approval changes (process and packaging changes), technology transfers, in addition to setting or extending shelf-life. Its versatility is illustrated with numerous examples for successful implementation in the development of monoclonal antibodies, fusion proteins, different types of vaccines, and in vitro diagnostic reagents. The verification of degradations models has been achieved by comparing the forecast with long-term experimental stability data establishing AKM as a reliable modeling approach. Though, it must be noted that adherence to best practices (‘good modeling practices’) is essential to obtain accurate results.

AKM appears as a reliable approach for stability modeling, shelf-life prediction, and many other matters during product development. It can effectively reduce development risks by making stability data available at a very early stage of development (stability forecasts) what otherwise, would be available only at the very end of a stability evaluation. In this relation, AKM should be encouraged as a routine practice to support the accelerated development of bioproducts.

## Supplementary Information

Supplementary Information.

## Data Availability

The datasets used and/or analysed during the current study are available from the corresponding author on reasonable request, except for B8 and V4 as they represent potentially proprietary information.
