# Supplementary material for: A universal tool for stability predictions of biotherapeutics, vaccines and in vitro diagnostic products
Source: Sci Rep. 2023 Jun 21;13:10077. doi: 10.1038/s41598-023-35870-6 (PMC10284933; doi:10.1038/s41598-023-35870-6)
Supplement: Supplementary file 1 — Supplementary Information. [file 41598_2023_35870_MOESM1_ESM.pdf]

A Universal Tool for Stability Predictions of Biotherapeutics, Vaccines and *In Vitro* Diagnostic Products

M. Huelsmeyer, D. Kuzman, M. Bončina, J. Martinez, C. Steinbrugger, J. Weusten, C. Calero-Rubio, W. Roche, B. Niederhaus, Y. VanHaelst, M. Hrynyk, P. Ballesta, H. Achard, S. Augusto, M. Guillois, C. Pszczolinski, M. Gerasimov, C. Neyra, D. Ponduri, S. Ramesh, D. Clénet

Supplementary information

Table S1. List of kinetic models.

Product / company	Stability attribute / Fig.	Kinetic model	Comment
B1 / Abbvie	Acidic isoform / Fig. 1.a	Model#1: $\frac{d\alpha}{dt} = 1.25 \times \exp(26.9) \times \exp\left(-\frac{118.0E3}{RT}\right) \times (1-\alpha_1)^2 - 0.25 \times \exp(20.2) \times \exp\left(-\frac{92.1E3}{RT}\right) \times (1-\alpha_2)^5$<br>Model#2: $\frac{d\alpha}{dt} = \exp(29.2) \times \exp\left(-\frac{117.9E3}{RT}\right) \times (1-\alpha)^{1.64}$	Model#1: wAIC / wBIC = 64.1% / 9.6%<br>Model#2: wAIC / wBIC = 35.9% / 90.4%
B2 / Novartis	Acidic variant / Fig. 1.b	$\frac{d\alpha}{dt} = \exp(27.5) \times \exp\left(-\frac{113.7E3}{RT}\right) \times (1-\alpha)^1$	
B3 / Sanofi	Asp isomerization / Fig. 1.c	$\frac{d\alpha}{dt} = 0.44 \times \exp(26.3) \times \exp\left(-\frac{109.8E3}{RT}\right) \times (1-\alpha_1)^4 + 0.56 \times \exp(20.3) \times \exp\left(-\frac{90.9E3}{RT}\right) \times (1-\alpha_2)^1$	
B4 / Sanofi	Charged isoform / Fig. 1.d	$\frac{d\alpha}{dt} = \exp(27.8) \times \exp\left(-\frac{114.7E3}{RT}\right) \times (1-\alpha)^1$	

B5 / Sanofi	Tg / Fig. 1.e	$\frac{d\alpha}{dt} = 0.10 \times \exp(28.6) \times \exp\left(-\frac{106.2E3}{RT}\right) \times (1-\alpha_1)^1 + 0.90 \times \exp(2.9) \times \exp\left(-\frac{54.7E3}{RT}\right) \times (1-\alpha_2)^1$	
B5 / Sanofi	Tg / Fig. 1.f	$\frac{d\alpha}{dt} = 0.99 \times \exp(10.9) \times \exp\left(-\frac{81.0E3}{RT}\right) \times (1-\alpha_1)^1 + 0.01 \times \exp(18.4) \times \exp\left(-\frac{81.2E3}{RT}\right) \times (1-\alpha_2)^1$	
B8 / MSD	Impurity / Fig. 1.g	$\frac{d\alpha}{dt} = \exp(19.5) \times \exp\left(-\frac{84.7E3}{RT}\right) \times (1-\alpha)^3 \times \alpha^2$	
B9 / Novartis	Impurity / Fig. 1.h	$\frac{d\alpha}{dt} = \exp(25.5) \times \exp\left(-\frac{107.5E3}{RT}\right)$	
B1 / Abbvie	HMW / Fig. 2.a	Model#1: $\frac{d\alpha}{dt} = \exp(22.2) \times \exp\left(-\frac{107.5E3}{RT}\right) \times (1-\alpha)^{14.7}$<br>Model#2: $\frac{d\alpha}{dt} = \exp(20.4) \times \exp\left(-\frac{103.0E3}{RT}\right) \times (1-\alpha)^{10}$	Model#1: wAIC / wBIC = 33.8% / 15.3%<br>Model#2: wAIC / wBIC = 23.0% / 52.3%
B7 / Sanofi	Dimer	$\frac{d\alpha}{dt} = 0.72 \times \exp(13.8) \times \exp\left(-\frac{78.8E3}{RT}\right) \times (1-\alpha_1)^1 \times C^{3.0} + 0.28 \times \exp(446.0) \times \exp\left(-\frac{114.7E3}{RT}\right) \times (1-\alpha_2)^1 \times C^{2.5}$	
B10 / Novartis	HMW / Fig. 2.c	$\frac{d\alpha}{dt} = \exp(18.7) \times \exp\left(-\frac{97.1E3}{RT}\right) \times (1-\alpha_1)^1$	
B11 / Novartis	HMW / Fig. 2.d	$\frac{d\alpha}{dt} = \exp(25.6) \times \exp\left(-\frac{118.4E3}{RT}\right) \times (1-\alpha)^1$	
V1 / Sanofi	Cell viability / Fig. 3.a	Model#1: $\frac{d\alpha}{dt} = 0.95 \times \exp(24.8) \times \exp\left(-\frac{90.4E3}{RT}\right) + 0.05 \times \exp(0.2) \times \exp\left(-\frac{30.9E3}{RT}\right) \times (1-\alpha_2)^1$<br>Model#2: $\frac{d\alpha}{dt} = \exp(13.0) \times \exp\left(-\frac{64.4E3}{RT}\right) \times (1-\alpha)^3$	Model#1: wAIC / wBIC = 74.7% / 1.0%<br>Model#2: wAIC / wBIC = 25.3% / 99.0%

V2 / Sanofi	Depolymerisation / Fig. 3.c	$\frac{d\alpha}{dt} = 0.91 \times \exp(28.8) \times \exp\left(-\frac{115.4E3}{RT}\right) \times (1-\alpha_1)^1$ $+ 0.09 \times \exp(6.3) \times \exp\left(-\frac{53.6E3}{RT}\right) \times (1-\alpha_2)^1$	
V3 / Sanofi	Antigen content / Fig. 3.e	Model#1: $\frac{d\alpha}{dt} = 0.14 \times \exp(41.5) \times \exp\left(-\frac{131.7E3}{RT}\right) \times (1-\alpha)^1$ $+0.86 \times \exp(237.4) \times \exp\left(-\frac{65.1E3}{RT}\right) \times (1-\alpha)^1$ Model#2: $\frac{d\alpha}{dt} = \exp(35.8) \times \exp\left(-\frac{122.8E3}{RT}\right) \times (1-\alpha)^{15}$	Model#1: wAIC / wBIC = 98.7% / 18.6% Model#2: wAIC / wBIC = 1.3% / 81.4%
D2 / bioMérieux	RFV / Fig. 4.a	$\frac{d\alpha}{dt} = 0.806 \times \exp(14.4) \times \exp\left(-\frac{77.3E3}{RT}\right) \times (1-\alpha_1)^1$ $+ 0.194 \times \exp(49.9) \times \exp\left(-\frac{164.7E3}{RT}\right)$	
D1 / bioMérieux	RFV / Fig. 4.b	$\frac{d\alpha}{dt} = \exp(14.0) \times \exp\left(-\frac{75.1E3}{RT}\right) \times (1-\alpha)^1 \times \alpha^{0.33}$ $+ \exp(85.8) \times \exp\left(-\frac{259.8E3}{RT}\right) \times (1-\alpha)^2$	
D3 / bioMérieux	RFV / Fig. 4.c	$\frac{d\alpha}{dt} = 0.84 \times \exp(52.3) \times \exp\left(-\frac{179.7E3}{RT}\right) \times (1-\alpha)^{0.7}$ $+ 0.16 \times \exp(11.6) \times \exp\left(-\frac{64.4E3}{RT}\right) \times (1-\alpha)^4$	
D4 / bioMérieux	RFV / Fig. 4.d	$\frac{d\alpha}{dt} = \exp(53.6) \times \exp\left(-\frac{180.3E3}{RT}\right) \times (1-\alpha)^{1.5} \times \alpha^{0.51}$ $+ \exp(5.7E-6) \times \exp\left(-\frac{46.6E3}{RT}\right) \times (1-\alpha)^2$	

ImmunoAssay *in vitro* Diagnostic

VIDAS® brand family is an automated solution of ELFA based immunoassays, offering more than 100 parameters. Every VIDAS® parameter kit is based on the interaction of two elements: the coated SPR receptacle, containing dried adsorbed antigens or antibodies, and the strip, made-up of a series of wells containing the correct amount of liquid reagents (alkaline-phosphatase immunoconjugate, washing solution, …) necessary for the test. Accuracy and reproducibility of results are insured using Standard and Control vials, consisting of different concentrations of the biomolecule of interest (liquid form or lyophilized).

The VIDAS® kit mechanism, relies on an immunologic reaction between mAbs and its targeted antigen(s), followed by an enzymatic revelation driven by the hydrolysis of 4-methylumbelliferyl phosphate (4-MUP) substrate by an alkaline-phosphatase immunoconjugate.

VIDAS® assay signal, known as Relative Fluorescence Value (RFV), is the representative result of all of the reactions involved during the different steps of the assay. This value, expressed as a ratio of $RFV_{day\ x}/RFV_{day\ 0}$, is used as reporting data for carrying out long-term Stability Predictions studies allowing batches comparison with potential initial offsets variabilities.

VIDAS® Cortisol S and VIDAS® PTH (1-84) kits stability prediction studies have been carried out using the whole kit compounds at different isotherms, except the only strip well containing the 4-MUP fluorescent substrate, subject to high hydrolysis rate with temperature increase, and generating too strong signal/noise for the VIDAS® instrument, set-up to prevent run launching in such condition.

VIDAS® NEPHROCHECK® kit is a biplex immunoassay detecting TIMP-2 (tissue inhibitor of metalloproteinases-2) and IGFBP-7 (insulin-like growth factor-binding protein 7) antigens. Both proteins are proposed as a mix in a single vial for standard and for control in this immunoassay kit.

Control and Standard vials are semi-finished reagents with own shelf-life, potentially different as of the full kit, and may be used from one kit batch to another by customers
